# Adapted Behavioural Activation for Bipolar Depression: A Randomised Multiple Baseline Case Series

**DOI:** 10.3390/brainsci12101407

**Published:** 2022-10-19

**Authors:** Kim Wright, Mohammod Mostazir, Ella Bailey, Barnaby D. Dunn, Heather O’Mahen, Michaela Sibsey, Zoe Thomas

**Affiliations:** 1 Department of Psychology, University of Exeter, Perry Road, Exeter EX4 4QG, UK; 2 Department of Psychology, University of Bath, Claverton Down, Bath BA2 7AY, UK; 3 School of Psychology and Clinical Language Sciences, University of Reading, Earley, Reading RG6 6DZ, UK; 4 Elizabeth Fry Building, School of Psychology, University of Surrey, Guildford GU2 7XH, UK

**Keywords:** bipolar disorder, bipolar depression, Behavioural Activation, psychological therapy

## Abstract

Behavioural Activation (BA) is associated with a substantial evidence base for treatment of acute unipolar depression, and has promise as an easily disseminable psychological intervention for bipolar depression. Using a randomised multiple baseline case series design we examined the feasibility and acceptability of an adapted version of BA in a U.K. outpatient sample of 12 adults with acute bipolar depression. Participants were allocated at random to a 3–8 week wait period before being offered up to 20 sessions of BA. They completed outcome measures at intake, pre- and post-treatment and weekly symptom measures across the study period. Retention in therapy was high (11/12 participants completed the target minimum number of sessions), and all participants returning acceptability measures reported high levels of satisfaction with the intervention. No therapy-related serious adverse events were reported, nor were there exacerbations in manic symptoms that were judged to be a result of the intervention. The pattern of change on outcome measures is consistent with the potential for clinical benefit; six of the nine participants with a stable baseline showed clinically significant improvement on the primary outcome measure. The findings suggest adapted BA for bipolar depression is a feasible and acceptable approach that merits further investigation.

## 1. Introduction

Bipolar I disorder has been estimated to affect around 1% of people, and bipolar II disorder around 1.6%, with many experiencing multiple episodes of mania/hypomania and depression across their lifetime [1,2]. Major depressive episodes, which are experienced by the majority of people with bipolar disorders (BD), can last from weeks to months or even years [3], can impact significantly upon work and relationships, and are associated with increased risk of suicide [4].

There are recommended pharmacological approaches for the treatment of bipolar depression for example [5,6]. However, not all individuals with bipolar depression want to take medication, or are able to tolerate it, for example because of side-effects or the need for frequent monitoring. Furthermore, few medications have consistent empirical support for their efficacy in the treatment of bipolar depression, and their long-term protective effect is largely uncertain [7].

Psychological therapies for depression have been developed and tested extensively amongst individuals with unipolar depression. Given the similarities between unipolar and bipolar depression, these offer promise as an adjunctive or alternative therapy for those with acute bipolar depression. Whilst there is evidence for a reduction in depressive symptoms amongst those receiving certain psychological therapies for BD in general [8] only a small number of studies have tested the efficacy of targeted psychological therapies for acute bipolar depression [9]. There is a need for further evaluation of credible and easily disseminable psychological interventions for this phase of BD.

Behavioural Activation (BA) is an established therapy for acute unipolar depression [10] that seeks to re-establish healthy patterns of activity and replace behaviours that function to avoid or manage distress with more adaptive behaviours that are rewarding, or constructive in the longer term [11]. An advantage of BA in unipolar depression is that is appears to be as effective as Cognitive Behavioural Therapy (CBT), whilst arguably being simpler to train and to deliver [12]. This has implications for the accessibility of psychological therapy for individuals with bipolar depression. Although less prevalent than unipolar depression, bipolar depression has a relatively high rate of recurrence; therefore, individuals may require support at a number of points across their lifetime, and at different points in the care system. Having a psychological treatment for this phase of bipolar disorder, which is relatively simple to learn and deliver, and can thus be delivered by a variety of workforces at different points in the care system, is likely to increase ease and timeliness of access to treatment. Whilst there is some overlap between unipolar and bipolar depression, it is nevertheless necessary to separately evaluate BA for the treatment of bipolar depression. In its simplest form, BA involves increasing levels of rewarding activity in depressed individuals, yet there is evidence to suggest that bipolar disorder may be associated with differences in reward processing by the brain [13], such that some people with the condition may be at risk of (hypo)mania if they engage in too much challenging or reward-striving activity. Therefore, adaptation of the approach, as well as evaluation of its safety, appears warranted for this population.

Very little research has investigated BA for bipolar depression. In a case series of 12 individuals in the United States, with acute bipolar depression, recruited through hospital settings, BA delivered in up to 16 sessions was found to be acceptable to patients, and the majority of participants showed clinically significant improvement in depression symptoms [14]. In the current study, we sought to build on this work by evaluating BA as an approach for people recruited from across primary and secondary care and in a U.K. healthcare context. We used case series methodology which provides a relatively rapid means of assessing treatment acceptability and feasibility as well as the opportunity to develop and refine the treatment protocol [15] and can also be used to provide a preliminary indication of the clinical promise of an intervention.

Our research aims were to evaluate if our protocol for Behavioural Activation for bipolar depression (BA-BD): (i) is feasible and acceptable to patients; (ii) results in at least minimally clinically significant improvement in depression symptoms for a majority of participants; (iii) does not result in significant adverse reactions for any participants. We set the following as minimal thresholds to be met in order to indicate likely acceptability and safety, and to proceed to further evaluation of the therapy: (a) >50% of clients complete treatment; (b) clients on average attend >50% of scheduled sessions; (c) no serious incidents (serious adverse events such as physical or mental health emergencies) occur that can be clearly attributed to the intervention; (d) >60% of clients show at least minimum clinically significant improvement in depression symptoms as measured by the Patient Health Questionnaire (PHQ-9 [16]); (e) <30% of clients show at least minimum clinically significant clinical deterioration on the PHQ-9.

The behavioural theory of depression predicts an inverse relationship between values-consistent activities and mood, stipulating that the inability to follow low mood with such behaviours is characteristic of depression. There is support for this hypothesis in unipolar depression [17] however it has not been tested in people with bipolar depression, nor has the relationship between manic symptoms and values-consistent behaviour been investigated. In the current dataset we tested the prediction that there would be a negative association within individuals across time between values-consistent behaviour and both depression and suffering. We also explored the association within individuals across time between manic symptoms and values-consistent behaviour.

## 2. Materials and Methods

The study was preregistered with ClinicalTrials.gov (NCT03658824). All participants gave their informed consent for inclusion before they participated in the study. The study was conducted in accordance with the Declaration of Helsinki, and the protocol was approved by the U.K. National Research Ethics Service (Ref: 18/SW/0116).

### 2.1. Design

This study used a two-wave randomised multiple baseline ABA case-series design. In an ABA design, for an individual receiving a treatment there is a measurement period (baseline) before the treatment phase, and then again after the treatment phase, to allow exploration of the stability of any changes observed over the treatment period. In a multiple baseline case series, individuals in the study are randomly allocated to baseline periods of different lengths—the timing of the start of treatment is staggered across participants. This design increases the likelihood that symptom change following the start of treatment is due to the effects of treatment rather than to non-treatment-related factors such as measurement repetition effects or spontaneous recovery. Where reliable improvement in symptoms is found from pre to post treatment, a randomised multiple baseline design increases confidence that this effect is a result of treatment rather than timing [18].

It is good practice to have at least three replications of the pattern of change across different cases (inter-case replication) to have at least six different lengths of baseline that individuals can be randomised to [19,20]. Therefore, in the current study, we aimed to randomise 12 participants to between three and eight weeks of baseline measurement (allowing a minimum of three weekly measurements during the baseline phase, and six baseline lengths to which the participants could be randomised). We delivered the intervention in two consecutive cohorts (*n* = 6 in each) to allow us to refine the therapy protocol between cohorts; in practice this occurred iteratively throughout the case series.

### 2.2. Participants

Participants were recruited from secondary care mental health services and through advertisements in the local community in Devon, U.K. between October 2018 and February 2020. Participants were required to be aged 18 or over and currently suffering from a major depressive episode (within the context of Bipolar I or II Disorder) as their primary presenting problem. In addition, the study required participants to: (i) score > 9 on a self-report measure of depression severity (the PHQ-9); (ii) meet diagnostic criteria for depression according to DSM-V [21]; (iii) meet diagnostic criteria for Bipolar I or II Disorder (DSM-V). Participants were required to have working knowledge of written and spoken English, sufficient to be able to make use of therapy and to be able to complete research assessments without the need of a translator as funding was not available for this.

Exclusion criteria were: (i) current/past learning disability, organic brain change, or substance dependence (drugs and alcohol) that would compromise ability to use therapy; (ii) current marked risk to self (i.e., severe and frequent self-harm, immediate risk of suicide) that could not be appropriately managed in a psychological therapies outpatient setting; (iii) currently lacking capacity to give informed consent; (iv) currently receiving other psychosocial therapy for depression or bipolar disorder; and (v) presence of another area of difficulty that the research team and participant believed should be the primary focus of intervention (for example, Post-Traumatic Stress Disorder, psychosis).

### 2.3. Intervention

Participants were offered up to 20 individual therapy sessions of Behavioural Activation (number determined collaboratively by therapist and participant according to progress and participant preference) of up to 60 min duration, plus one booster session three months after the end of therapy. Sessions were supplemented by home practice and were delivered either face-to-face or by telephone/online due to the onset of COVID-19 restrictions midway through the study.

The COBRA BA protocol was adapted to better address the needs of individuals with Bipolar Depression. This included consideration of the patient’s relationship to medication and their access to information about Bipolar Disorder. To address concerns about (hypo)manic relapse we included contracting work with the client at an early stage about how symptoms of hypomania/mania would be noticed and responded to within therapy. We also used the framework of functional analysis to explore mood-driven behaviour prompted by positive, angry or energised states rather than only by negative feelings, and consequently to explore alternative behaviours. When considering new behaviours, we adapted the process of scheduling of positive activities to (i) discriminate between activities that promote hypomanic/manic states, and those that promote less “risky” positive states; (ii) increase emphasis on an appropriate balance of rest and activity; (iii) increase emphasis on exploring the role of daily routine in symptom maintenance. Finally, we extended relapse prevention work to take into account manic as well as depressive relapse. As was the case in the COBRA trial, participants could bring a friend or family member to sessions if wished.

Therapy was delivered by two experienced psychological therapists with specialist training in Cognitive and Behavioural Therapies. One therapist was the lead developer of the BA-B protocol (KW); the other received additional training in the approach. Therapist one treated four clients (two per wave) and therapist two treated eight clients (four per wave). Therapy was delivered in an outpatient psychological therapies service. In terms of the healthcare context, participants held in secondary care typically had access to medication reviews with a psychiatrist, and in some cases regular meetings with a mental health professional that did not involve formal psychological therapy. Participants held in primary care had access to meetings with their primary care physician for mental health care purposes, including medication.

### 2.4. Outcomes

Feasibility and Acceptability. Numbers of patients expressing interest, consented, assessed, eligible, randomised and completing outcome measures were used to assess feasibility and acceptability, as was participant attrition from the trial and from treatment. To assess continuation criteria, we inspected proportion of participants completing treatment and attending at least the minimum number of scheduled sessions. Acceptability of the treatment and research procedures was also assessed using six items rated on a five-point Likert scale (e.g., “Overall, how satisfied were you with the BA-BD programme? Not at all satisfied” to “Extremely satisfied) (see Supplementary Materials). Participants could also provide open-ended written responses about helpful/unhelpful aspects of treatment, barriers to and impact of therapy, and experiences of each stage of the research (All participants were also invited to take part in a semi-structured interview, which asked about their experience of the trial and therapy. Analysis of qualitative feedback from participants will be reported separately). Treatment attendance was recorded, with treatment completion defined as attending at least eight therapy sessions [12].

Therapy Safety. Adverse events, including serious adverse events, were monitored and recorded throughout the study. Adverse events were defined as any untoward or unintended medical occurrence or response, whether causally related to the trial treatments or not. Serious adverse events were those which were fatal, life threatening, resulted in or prolonged hospitalisation, resulted in significant disability or incapacity or any condition judged significant by a clinician. Serious adverse events were reviewed by two independent clinicians for study or therapy relatedness in order to provide information on therapy safety. Given concerns about manic “switching” resulting from exposure to certain antidepressant somatic interventions [22] and also the theoretical concerns about the activating effects of challenging or rewarding activities outlined previously, we also report reliable deterioration on an objective measure of manic symptoms, pre to post therapy.

Weekly Measures. Each week during the baseline, therapy and follow-up periods participants were asked to complete: the Patient Health Questionnaire-9 (PHQ-9 [16]), a nine-item self-report measure of depressive symptoms; the Altman Self-Rating Mania Scale [23], a five-item self-report measure of hypomania symptoms over the past week; and three self-report questions about suffering, struggling and engagement in valued activities [24]. These three questions (henceforth referred to as SSVA questions) are rated on a scale from 0 (not at all) to 10 (extreme amount). They address core hypothesised process of change in behavioural activation, namely reduction in avoidance behaviours (i.e., reduction in struggling in the face of suffering) and increase in rewarding behaviours (i.e., engagement in valued activities (A number of participants reported difficulty in comprehending and answering the weekly “struggling” question. As this raised concerns about its validity in this sample, prior to commencing analysis, the decision was made to exclude it)).

Other Clinical Outcome Measures. In addition the following measures were completed at study intake, pre-therapy and post-therapy: the Hamilton Depression Scale [25], a 17-item observer-rated scale measuring symptoms of depression over the past week; the Bech–Rafaelsen Mania Scale (BRMS [26]), an 11-item observer-rated scale measuring the severity of manic states; the Work and Social Adjustment Scale (WSAS [27]), a 5-item self-report scale of functional impairment attributable to an identified problem; the Brief Quality of Life in Bipolar Disorder (Brief QoLBD [28]), a 12-item self-report measure of disorder-specific quality of life; the Beck Depression Inventory [29], a 21-item self-report measure of depressive symptoms and attitudes; the General Anxiety Disorder Assessment-7 (GAD-7 [30]), a 7-item self-report measure of anxiety symptoms; the Short Warwick–Edinburgh Mental Well-being scale (SWEMWBS [31]), a 7-item self-report measure of wellbeing; the Snaith–Hamilton Pleasure Scale (SHAPS [32]), a 14-item self-report measure of level of anhedonia.

Process Measures. The following measures were completed at study intake, pre- and post-therapy to assess change in key processes hypothesised to be addressed by Behavioural Activation: the Behavioral Activation for Depression Scale (BADS [33]), a 25-item self-report measure of changes in activation and avoidance over the past week; the Positive and Negative Urgency Subscales of the UPPS-P Impulsive Behavior Scale [34] (14 and 12 items, respectively), measuring tendency to respond impulsively to positive or negative feelings. This measure was included because BA aims to weaken the link between strong negative mood and an impulsive behavioural response (avoidance).

### 2.5. Procedure

Following initial contact with the research team, and having been sent the Participant Information Sheet, participants attended an intake assessment interview in person with a member of the research team where written, informed consent was obtained. Consenting participants were then asked for demographic information and assessed for eligibility using the mood disorders, psychosis screening and substance dependence sections of the SCID-5 [35] (The SCID-IV (1996) was used to assess presence of substance dependence), the PHQ-9, and through additional questioning, for example about the participant’s primary presenting difficulty and current engagement in other psychological therapy. Eligible participants completed the WSAS, GAD-7, ASRM, BDI, QoLBD, BADS, SWEMWBS, SHAPS and UPPS-P. The BRMS and HAM-D observer-rated measures were completed by the researcher.

Participants were then randomised to one of six wait periods (three, four, five, six, seven or eight weeks). Starting one week after the baseline assessment participants completed weekly symptom measures (PHQ-9, ASRM) and questions about suffering, struggling and engagement in valued activities over the wait period, therapy period and for three weeks post therapy. These were completed online or on paper via post.

After reaching the end of their allocated wait period, participants completed the WSAS, GAD-7, BDI, QoLBD, BADS, WEMWEBS, SHAPS and UPPS-P, as well as the HAM-D, Bech and current depressive episode section of the SCID. Therapy then commenced on an approximately weekly basis. At the end of therapy participants completed the set of measures completed pre-therapy once again, and were invited to take part in the semi-structured interview. The order of measures completed is displayed in Table 1.

The end of the study (October 2020) was defined as the final piece of data being collected from the final participant.

#### Randomisation, Concealment of Allocation, and Blinding

Participants were allocated to wait periods by a researcher independent of the study team, using the random number generator function within Microsoft Excel. The research team were not blinded to participants’ allocations because allocation determined the schedule of assessments required prior to therapy starting.

### 2.6. Data Analysis

Quantitative feasibility, acceptability and safety data and aggregate scores on clinical process and outcome measures are presented descriptively. With regard to assessing change in clinical outcome measures we calculated proportion of participants showing minimum clinically significant improvement or deterioration on the PHQ-9 (primary outcome measure) in order to address threshold rules (d) and (e). We defined this as a change in PHQ-9 score of at least 6 points, following an established standard in benchmarking therapies for unipolar depression [36]. We also examined both reliable change and reliable and clinically significant change [37] between the pre-therapy and post-therapy assessments, using the Leeds Reliable Change Calculator [38]. Reliable change was defined as a change in score greater than 1.96 the standard error of the difference between the pre- and post-therapy scores. Clinically significant change was defined as the participant showing both a reliable improvement in the score on a given measure, and falling below the clinical cut-off on that measure post-treatment. Where published cut-offs were not available, we used scale validation papers to derive comparison sample values and defined clinically significant change using Jacobson et al. [39] criterion B or C, depending on which comparative norms were available. We report the number of participants showing both reliable change and reliable and clinically significant change on each measure, as well as the overall effect size of the change on each measure. These calculations were conducted only for those participants showing a stable baseline in terms of depression as measured by the PHQ-9. Unstable baseline was defined as a decrease in PHQ-9 score of at least six points and a score of less than 10 at pre-treatment.

As part of our exploratory analyses, we examined the overall change in weekly scores on the PHQ-9, the ASRM and the SSVA questions as a function of time. The models used did not require a stable baseline period; thus, all 12 participants could be included. Multi-level random intercept/coefficient models were developed separately for each of the four outcome variables. Subject-specific time-related slopes were included to capture differential effect of time among subjects. We carried out log likelihood ratio (LR) tests to test whether subject specific effect of time was different than the overall time effect. Next, using the same multi-level modelling approach, we examined associations between the four variables with separate models for each pair of variables.

## 3. Results

**Figure 1:**
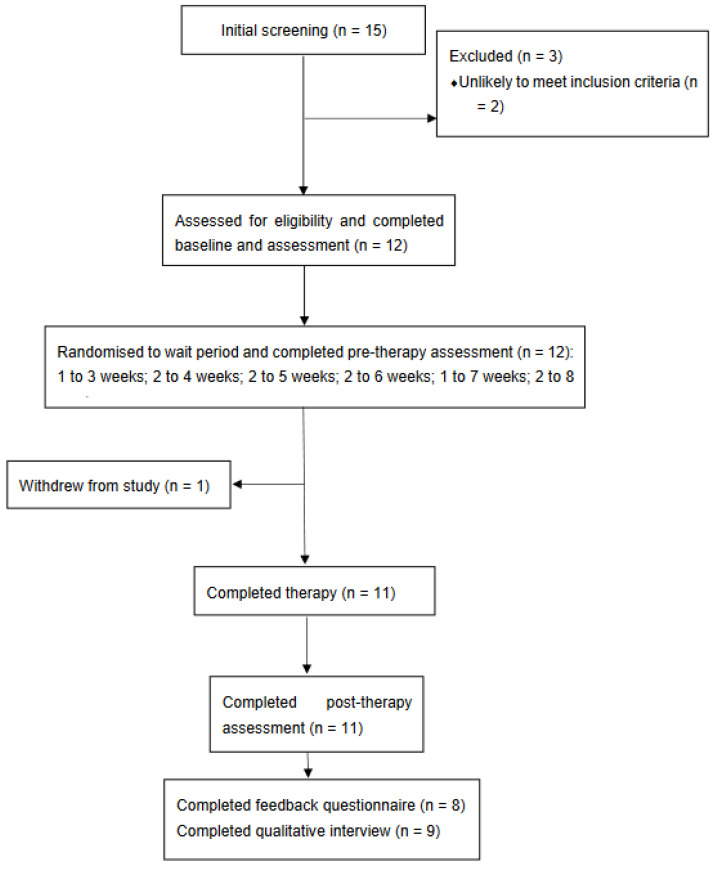
CONSORT diagram showing participant flow.

Figure 1 displays the Consort diagram for the study. A total of 38 individuals expressed an interest in taking part in the study. Of these, 15 completed a telephone screening call with study researchers; 12 of these went on to complete the eligibility assessment and were found to meet study entry criteria. Randomisation resulted in three participants being allocated to three weeks wait, two to each of four, five, six and eight weeks wait, and one to seven weeks wait.

Of the 12 participants randomised, one withdrew from the study after receiving seven sessions of therapy; the reason given was the impact of the onset of the COVID-19 pandemic upon their situation. Eleven participants thus completed the post-treatment assessment and the three-week post-treatment monitoring period. Of these 11, two did not have a stable pre-treatment baseline and so were not included in the analyses of reliable change.

Table 2 displays demographic and clinical characteristics of the participants at study intake. The majority of the sample were female and all were white British, with a median age of 44 (range 31 to 65). Five were receiving care from secondary mental health services on an outpatient basis; the remainder were receiving their mental health care from their primary care physician.

The mean duration of participants’ current depressive episode was 7.58 weeks, SD = 8.89, and participants reported having a median of 35 previous episodes, range = 5 to 120. Ten participants were prescribed psychiatric medication at the point of study entry; of these six were prescribed a mood stabilising medication, seven an antipsychotic medication and five an antidepressant.

Table 3 displays mean scores on other measures conducted at study intake. Self-reported depression scores were in the severe range, those clinician-rated mood (HAM-D) was in the moderate range. Participants also had significant self-reported anxiety symptoms, with mean ratings in the moderate range. They self-reported manic symptoms in the non-clinical range. Mean quality of life (QoL.BD) was around one standard deviation below that of a reference sample of individuals with Bipolar Disorder [28].

### 3.1. Acceptability and Feasibility

As can be seen on the CONSORT diagram, nine participants completed all 20 sessions. One participant completed after 13 sessions due to therapist and participant agreeing they were in sustained recovery, one participant missed multiple sessions due to illness therefore completed after 14 sessions, and as previously mentioned one opted to end therapy and study involvement after attending seven sessions. In total therefore, 11 of the 12 participants completed therapy (defined as attending at least eight therapy sessions). Ten of these participants opted to attend at least two of the possible three booster sessions; the remaining participant reported not needing the booster sessions. Participants attended a median of 91% (range: 57–100%).

100% of scheduled sessions, the remainder being either cancelled by the patient or missed without notice.

Eight participants returned the acceptability survey. Seven (88%) participants reported finding the therapy aspects of the study “very” or “extremely” acceptable (approach and activities made sense and were reasonable) with one finding them “moderately” acceptable. All eight participants were “very” or “extremely” satisfied with the therapy and would be “likely” or “very likely” to recommend it to a friend.

### 3.2. Therapy and Study Safety

Over the course of the study, seven participants reported a total of 13 adverse events. Of these, four were serious adverse events: one participant attempted to end their life on one occasion and was admitted to hospital due to psychiatric symptoms on two further occasions; a second participant was admitted to hospital for psychiatric symptoms on one occasion. All four events were judged to be unrelated to participation in the study or receipt of the therapy and both participants chose to continue to attend therapy sessions.

### 3.3. Change in Clinical Outcome Measures

Table 3 displays aggregated mean scores and effect sizes for the clinical outcome measures, whilst Table 4 shows the pattern of reliable and reliable and clinically significant change across participants.

Of the nine participants whose scores were stable across the baseline phase, seven showed reliable improvement from pre to post treatment, one showed reliable deterioration and one no change on the PHQ-9. Of the seven showing reliable change, six also met criteria for clinically significant improvement and were in remission according to their PHQ-9 score (score < 10). Relevant to threshold rules (d) and (e), all seven showed a decrease in PHQ-9 score of at least six points and one participant showed an increase of at least six points.

Our secondary measures of depression were the BDI and HAM-D. On the BDI five participants showed reliable improvement (which was also clinically significant for three) and the remainder no change. Two showed reliable and clinically significant improvement and one reliable deterioration on the HAM-D. Levels of mania symptoms, measured by the BMRS, were relatively low at intake, and with one exception these remained low at post-treatment.

In terms of change in anxiety, one participant showed reliable deterioration and six showed reliable improvement (five of whom showed clinically significant improvement).

On both the quality-of-life measure (QoL.BD) and the daily functioning measure (WSAS) no participants showed reliable deterioration. Five showed reliable improvement on the QoL.BD and six showed reliable improvement on the WSAS (in one this was also clinically significant).

On the wellbeing measure (WEMWBS) five showed reliable improvement (also clinically significant in four), with one showing reliable deterioration. Anhedonia levels, as measured by the SHAPs, were high at pre-treatment and showed reliable improvement in four participants (clinically significant in two) with the remainder showing no change.

On measures of process, over half of the sample (five) showed reliable improvement in self-reported negative urgency whilst two showed reliable improvement in self-reported positive urgency. Five showed reliable improvement in avoidance (also clinically significant in four). None showed reliable deterioration in any of these measures.

All instances but one of reliable deterioration post treatment and all but one of the serious adverse events were in relation to one participant. This was not judged to be a consequence of therapy attendance.

### 3.4. Exploratory Analysis of Change in PHQ9, ASRM, Suffering and Valued Action over Time

In the analyses that follow, data from all 12 participants were included, as the models used do not require a stable baseline period to explore overall pattern of change across time. Using multilevel models, we first examined change in each of the four variables over time for each of the four variables. Subject-specific random intercepts models were fitted followed by models with random time-related slopes. Non-significant log likelihood ratio test statistics indicated running random intercept models rather than random coefficient models for all the variables. Over the weeks, depression (PHQ-9) and suffering showed a significant downward trajectory (PHQ-9: −0.40, 95% CI: −0.48 to −0.32, *p* < 0.001; Suffering: −0.07, 95% CI: −0.11 to −0.04, *p* < 0.001), valued action showed a significant increase over time (Valued action: 0.07, 95% CI: 0.04 to 0.10, *p* < 0.001), and mania scores (ASRM) showed an increase in marginal significance (Mania: 0.05, 95% CI: −0.00 to 0.10, *p* = 0.05).

### 3.5. Relationships between PHQ-9, ASRM, Suffering and Valued Action over Time

For each variable pair of interest (PHQ-9 and valued action, suffering and valued action, mania and valued action) we examined the association between them over time. Table 5 displays the results of the three models.

**Figure 2:**
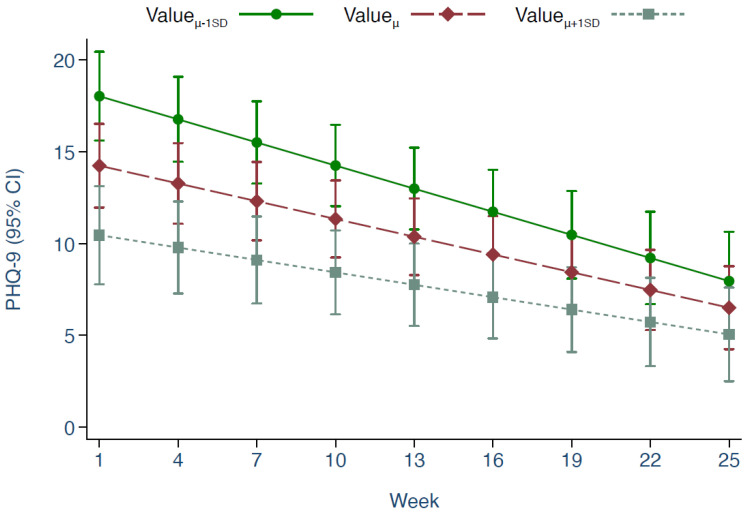
PHQ-9 predicted by valued action. Note. Lines depict estimated PHQ-9 scores at mean, and 1 SD above and below, of valued action for the sample over the study period; error bars depict 95% CIs of the estimates.

To explore the associations between pair of variables of interest, we predicted PHQ-9, suffering and mania by valued action after controlling the model for time (Table 4). Higher valued action was associated with lower PHQ-9 (*β* = −1.29, 95% CI: −1.69 to −0.89, *p* < 0.001), which was qualified by a significant ‘timeXvalued-action’ interaction (*p* < 0.01) indicating differential effect of valued action on PHQ-9 over time. To explore the moderated relationship, we predicted PHQ-9 over time at different levels of valued action (mean, below 1SD, and above 1SD) (Figure 2). Patients with a valued action score below 1SD at the beginning of the study (week-1) showed greater improvement in their PHQ-9 score at the end (week-25) compared with those who were above 1SD at the beginning (Δ- 4.67, 95% CI: −8.38 to −0.95, *p* = 0.01). There was no moderation by time of the relationship between suffering and valued action (*p* = 0.31) but their association was negative (−0.45, 95% CI: −0.58 to −0.32, *p* < 0.001). We found mania and valued action to be positively associated (0.49, 95% CI: 0.31 to 0.67, *p* < 0.001). This association remained positive (*p* = 0.001) when we further adjusted the model by including PHQ-9 and suffering scores. The interaction between valued action and time was non-significant (*p* = 0.61) suggesting a similar positive association over time.

## 4. Discussion

This case series supports the feasibility and acceptability of adapted BA for bipolar depression. Our feasibility targets were met and exceeded. We defined feasibility as participants on average attending more than half of the sessions that were arranged with them and more than half completing treatment. We found that all but one participant completed treatment: further, quantitative participant feedback indicated high levels of satisfaction with the treatment, as well as acceptability of the approach and activities involved. There were no serious incidents that could be attributed to the intervention, meeting our third feasibility criteria. Despite it being likely that scores on measures of mania symptoms might increase slightly as depression diminishes due to improvements in sleep, mood and activity for example, there were no instances of mania that were judged to be a consequence of the therapy, and only one participant showed reliable deterioration in mania symptoms at the end of treatment.

Finally, both of our clinical outcome criteria were met. We delineated that over 60% of participants should have minimal clinically significant improvement on the Patient Health Questionnaire (PHQ-9 [16]), and fewer than 30% of clients should show clinically significant clinical deterioration on the PHQ-9. We found 78% showed minimal clinically significant improvement in PHQ-9 score from pre- to post- treatment, and only one participant (11%) showed minimal clinically significant deterioration. The overall pattern of change on the clinical outcome measures supports the potential of the approach to confer clinical benefit.

These findings provide further support demonstrating the potential of BA for people with bipolar depression [14], extending this to a sample of individuals who were not receiving ongoing psychiatric support in secondary care. This is important because high thresholds for entry into secondary care in some healthcare systems can mean that many people with bipolar depression are being supported by their primary care physician only. BA is relatively easily trainable; therefore, it represents an approach that could potentially be delivered at primary care level.

In terms of findings relevant to the mechanism of BA for bipolar depression, the pattern of change in mood-related impulsivity (negative urgency) and self-reported avoidance behaviour was consistent with the possibility that adapted BA acts on these processes. Additionally, pertinent to therapy process, we explored relationships between depression and hypomania symptoms and approach behaviours (here conceptualised as actions consistent with values), within participants across the course of therapy. As expected, higher participant-reported engagement in values-consistent behaviour was associated with both lower depression symptoms and lower participant suffering. This is consistent with behavioural accounts of depression, which propose an inverse relationship between low mood and engagement in positively reinforced behaviour. Over time, relative to those with high levels, those with low levels of valued action showed a greater decrease in depressive symptoms. Without a comparator group who did not receive the intervention, changes over time cannot be attributed to the effect of the intervention. Furthermore, these results may represent ceiling and floor effects, or the effects of spontaneous recovery, whereby those with higher levels of depression and lower levels of valued action had greater capacity to show change over time. Nevertheless, our findings contribute to a novel area of investigation: testing the model underpinning behavioural therapy for bipolar depression. According to this model, mood and valued action should be inversely correlated within individuals; hence, behavioural therapy encourages engagement in valued action despite low mood. Our findings support the presence of this inverse relationship in our sample and illustrates the potential to explore within-participant inter-relationships between process and outcome variables in behavioural therapy for bipolar depression. It should be noted however that our study had a small sample size hence estimates are imprecise. Studies using larger samples are needed, whilst inclusion of a comparator group would allow investigation of whether changes over time are likely to be a result of therapy.

Our study allowed us to explore a novel area of investigation: the relationship between hypomanic symptoms over time with engagement in valued action. In our sample these were positively associated, such that greater valued action was associated with greater levels of hypomanic symptoms. This association remained when levels of depression were taken into account, suggesting the relationship is not simply reflective of levels of concurrent depressive symptoms. Our data do not permit exploration of the causal relationship between hypomania and valued action; however, in future developments of behavioural activation for bipolar depression, it will be valuable to know whether the enhancement of values-consistent activity carries a risk of exacerbating hypomanic symptoms, and if so, how this can be mitigated. In the current study, we mitigated the theoretical risk by monitoring hypomanic symptoms carefully in therapy sessions and by promoting balanced activity and rest, rather than encouraging clients to maximise levels of activity and goal-striving, and there were no instances where increases in manic symptoms were judged to be due to the therapy. Again, these findings are preliminary and from a small sample. Future studies using larger samples and measurement points that are spaced similarly across participants would allow examination of cross-lagged associations between depressive and hypomanic symptoms and engagement in valued activity across time using structural equation modelling. This would permit tests of hypothesis regarding the extent to which valued action temporally predicts change in bipolar symptoms, and vice versa.

Strengths of the study overall include diagnosis of bipolar disorder using a replicable method involving a validated, observer-rated instrument, and use of a randomised, multiple-baseline design which increases confidence that pre- to post- treatment symptom change may be related to the intervention rather than the passage of time. Our study has a number of limitations. Generalisability of our findings pertaining to feasibility and acceptability is limited by the fact it was conducted at a single site with only two therapists (with a relatively high level of experience and training), in an ethnically homogenous U.K. sample, meaning that we were unable to gather data on acceptability cross-culturally. We also did not receive feedback on therapy acceptability from all participants; it is possible those with negative experiences are less likely to provide such feedback thus therapy acceptability may be overstated.

With regard to examining potential efficacy of the intervention, inherent limitations of this design include the inability to draw conclusions about causality due to the absence of a comparator arm. Randomised controlled trials are necessary to establish the effect of the intervention. In addition, we note the marked variability in depression and mania symptom levels exhibited by some participants across the study period. This is characteristic of a subgroup of people with bipolar disorder [40] and presents a challenge to the practice of evaluating depression outcome in this population through the use of a measure at a single end point. Consequently, for those measures not gathered weekly, the stability of any improvement cannot be characterised. Alternative practices may include assessing stability as well as level of symptoms [41], calculating the mean symptom level over period of time post-intervention, and specifying co-primary outcome measures addressing sense of recovery or daily functioning (for example [42]).

In conclusion, in our sample, we found our BA protocol for bipolar depression to be safe, feasible and acceptable. The pattern of change on the clinical outcome measures is consistent with the potential for clinical benefit. These findings, in conjunction with the extant literature evaluating BA within unipolar depression, provides proof-of-concept for BA as an intervention worthy of further evaluation in this population.

## Figures and Tables

**Table 1:** Measures completed at each stage of the study.

Measure	Intake	Pre-Therapy	Weekly	Post-Therapy
SCID-5	X	X		X
PHQ-9	X		X	
ASRM			X	
SSVA			X	
BDI	X	X		X
HAM-D	X	X		X
BMRS	X	X		X
GAD-7	X	X		X
WEMWBS	X	X		X
WSAS	X	X		X
QoL.BD	X	X		X
SHAPS	X	X		X
NU	X	X		X
PU	X	X		X
BADS	X	X		X

Note. SCID-V = structured clinical interview for DSM-V (selected sections); PHQ-9 = Patient Health Questionnaire-9; ASRM: Altman Scale for Rating Mania; SSVA = suffering, struggling and valued action questions; BDI = Beck Depression Inventory; HAM-D = Hamilton Depression Rating Scale; BMRS = Bech Mania Rating Scale; GAD-7 = Generalised Anxiety Disorder Assessment; WEMWBS = Warwick–Edinburgh Mental Wellbeing Scale; QoL.BD = Quality of Life in Bipolar Disorder Scale; SHAPS = Snaith–Hamilton Pleasure Scale; NU = Negative Urgency subscale; PU = Positive Urgency subscale; BADS = Behavioural Activation for Depression Scale; X indicates measure was completed at this timepoint.

**Table 2:** Clinical and demographic characteristics of participants.

Ppt	Gender	Age Group	Research Diagnosis	Current Medication	PHQ-9 Score at Intake
1	F	30–50	BD 1	Yes	23
2	F	50–70	BD 2	Yes	27
3	F	50–70	BD 1	Yes	17
4	F	30–50	BD 1	Yes	17
5	M	50–70	BD 2	No	12
6	M	30–50	BD 1	Yes	18
7	M	30–50	BD 1	Yes	26
8	F	30–50	BD 1	Yes	24
9	F	30–50	BD 2	No	23
10	F	30–50	BD 1	Yes	24
11	F	50–70	BD 1	Yes	24
12	F	30–50	BD 1	Yes	11

Note. Ppt = participant; F = female; M = male; BD 1 = Bipolar I Disorder; BD 2 = Bipolar 2 Disorder; current medication refers to current use of mood stabilising, antidepressant or antipsychotic medications.

**Table 3:** Mean (SD) and proportion showing reliable and clinically significant change for outcome measures over the study period.

Measure	Mean (SD) at Intake (*n* = 12)	Mean (SD) at Intake (*n* = 9)	Mean (SD) Pre-Treatment (*n* = 9)	Mean (SD) Post-Treatment (*n* = 9)	Mean (SD) Change Pre-Post	Cohen’s d (95% CI)
PHQ-9	20.50 (5.35)	21.11 (4.81)	16.44 (2.65)	9.11 (7.18)	7.33 (7.16)	1.02 (0.19, 1.82)
BDI	25.50 (13.50)	30.33 (10.23)	26.14 (7.89)	14.91 (11.80)	8.37 (8.13)	1.03 (0.19, 1.83)
HAM-D	16.19 (5.84)	18.03 (5.15)	12.97 (4.14)	11.48 (8.52)	1.49 (7.43)	0.20 (−0.47, 0.86)
BMRS	1.33 (2.39)	0.78 (1.30)	2.22 (2.33)	2.89 (2.85)	−0.67 (3.94)	−0.17 (−0.82, 0.49)
GAD-7	9.50 (3.96)	10.56 (3.00)	11.89 (3.33)	7.11 (4.54)	4.78 (5.07)	0.94 (0.13, 1.72)
WEMWBS	14.15 (4.56)	14.19 (4.31)	16.21 (2.66)	19.31 (4.79)	−4.22 (5.56)	−0.76 (−1.49, 0.007)
WSAS	26.42 (9.15)	28.44 (8.97)	30.78 (6.10)	22.05 (8.15)	6.72 (6.50)	1.04 (0.28, 1.76)
QoL.BD	27.58 (10.56)	26.56 (8.95)	27.78 (7.31)	37.22 (8.03)	−9.44 (6.21)	−1.52 (−2.48, −0.52)
SHAPS	34.78 (10.63)	37.15 (10.34)	33.44 (4.13)	29.69 (6.97)	3.75 (4.64)	0.81 (0.03, 1.55)
NU	2.96 (0.69)	3.18 (0.45)	3.45 (0.42)	3.10 (0.37)	0.35 (0.26)	1.36 (0.41, 2.26)
PU	2.81 (0.81)	3.11 (0.55)	3.19 (0.75)	2.99 (0.59)	0.20 (0.36)	0.57 (−0.15, 1.26)
BADS	58.00 (25.47)	51.89 (21.53)	55.44 (25.00)	82.48 (28.89)	−27.03 (21.46)	−1.26 (−2.13, −0.35)

Note. PHQ-9 = Patient Health Questionnaire-9; BDI = Beck Depression Inventory; HAM-D = Hamilton Depression Rating Scale; BMRS = Bech Mania Rating Scale; GAD-7 = Generalised Anxiety Disorder Assessment; WEMWBS = Warwick–Edinburgh Mental Wellbeing Scale; WSAS = Work and Social Adjustment Scale; QoL.BD = Quality of Life in Bipolar Disorder Scale; SHAPS = Snaith–Hamilton Pleasure Scale; NU = Negative Urgency subscale; PU = Positive Urgency subscale; BADS = Behavioural Activation for Depression Scale.

**Table 4:** Pattern of reliable and reliable and clinically significant change across those participants showing a stable baseline (*n* = 9).

Ppt No. Measure *	1	3	6	7	8	9	10	11	12
PHQ-9 ^E^	X	X			X		X	X	X
BDI ^E^		X				X			X
HAM-D ^E^		X		X					
BMRS ^E^	X	X							
GAD-7 ^E^		X				X	X	X	X
WEMWBS ^B^		X		X	X	X			
WSAS ^E^						X			
QoL.BD ^N^									
SHAPS ^B^		X		X					
NU ^C^									
PU ^C^									
BADS ^B^	X	X		X		X			

Note. PHQ-9 = Patient Health Questionnaire-9; BDI = Beck Depression Inventory; HAM-D = Hamilton Depression Rating Scale; BMRS = Bech Mania Rating Scale; GAD-7 = Generalised Anxiety Disorder Assessment; WEMWBS = Warwick–Edinburgh Mental Wellbeing Scale; QoL.BD = Quality of Life in Bipolar Disorder Scale; SHAPS = Snaith–Hamilton Pleasure Scale; NU = Negative Urgency subscale; PU = Positive Urgency subscale; BADS = Behavioural Activation for Depression Scale. Green = reliable improvement; blue = no change; red = reliable deterioration; X = reliable and clinically significant improvement; * superscript letters after measure names depict criterion used to evaluate clinically significant change [39]: B = criterion B, C = criterion C; E = external criterion; N = insufficient reference data available.

**Table 5:** Models for PHQ-9, Suffering and Mania predicted by value action.

Variables	Model: PHQ-9	Model: Suffering	Model: Mania
	Coef (95% CI)	Coef (95% CI)	Coef (95% CI)
Week	−0.52 (−0.68 to −0.35) ***	−0.04 (−0.08 to −0.00) *	0.02 (−0.03 to 0.07)
Valued action	−1.29 (−1.69 to −0.89) ***	−0.45 (−0.58 to −0.32) ***	0.49 (0.31 to 0.67) ***
WeekXvalued action	0.03 (0.01 to 0.06) *	-	-

Note. * denotes *p* < 0.05; *** *p* < 0.001.

## Data Availability

The data presented in this study are available on request from the corresponding author provided that suitable approvals are in place and the data can be shared anonymously. The data are not publicly available due to the need to preserve the anonymity of participants.

## Supplementary Materials

The following supporting information can be downloaded at: https://www.mdpi.com/article/10.3390/brainsci12101407/s1.
