# Supplementary material for: Adapted Behavioural Activation for Bipolar Depression: A Randomised Multiple Baseline Case Series
Source: Brain Sci. 2022 Oct 19;12(10):1407. doi: 10.3390/brainsci12101407 (PMC9599144; doi:10.3390/brainsci12101407)
Supplement: Supplementary file 1 [file brainsci-12-01407-s001.zip › brainsci-1944810-supplementary.pdf]

Supplementary Material

Acceptability feedback survey

Overall, how acceptable was BA-BD to you? In other words did you think that the treatment approach and activities made sense and were reasonable?

1	2	3	4	5
Not at all acceptable	Slightly acceptable	Moderately acceptable	Very acceptable	Extremely acceptable

Overall, how satisfied were you with the BA-BD programme?

1	2	3	4	5
Not at all satisfied	Slightly satisfied	Moderately satisfied	Very satisfied	Extremely satisfied

How likely would you be to recommend BA-BD to friends or family if they needed similar care or treatment?

1	2	3	4	5
Extremely unlikely	Unlikely	Neither Likely or Unlikely	Likely	Extremely Likely

Overall, how acceptable were the research aspects of the study to you? In other words did you think that what you were asked to do in terms of research assessments made sense and was reasonable?

1	2	3	4	5
Not at all acceptable	Slightly acceptable	Moderately acceptable	Very acceptable	Extremely acceptable

Overall, how satisfied were you with the research aspects of the study?

1	2	3	4	5
Not at all satisfied	Slightly satisfied	Moderately satisfied	Very satisfied	Extremely satisfied

How likely would you be to recommend taking part in a study like this to friends or family if they were eligible?

1	2	3	4	5
Extremely unlikely	Unlikely	Neither Likely or Unlikely	Likely	Extremely Likely
